# Prebiotic potential of proso millet and quinoa: Effects on gut microbiota composition and functional metabolic pathways

**DOI:** 10.71150/jm.2503002

**Published:** 2025-07-31

**Authors:** Jinwoo Kim, Jiwoon Kim, Yewon Jung, Gyungcheon Kim, Seongok Kim, Hakdong Shin

**Affiliations:** 1 Department of Food Science and Biotechnology, College of Life Science, Sejong University, Seoul 05006, Republic of Korea; 2 Carbohydrate Bioproduct Research Center, Sejong University, Seoul 05006, Republic of Korea

**Keywords:** gut microbiota, prebiotics, whole grain, proso millet, quinoa

## Abstract

Prebiotics are indigestible dietary components that improve host health by stimulating the growth and metabolic activity of beneficial intestinal microbes. The whole grains are rich in non-digestible carbohydrates, which may confer prebiotic potential. Among them, millet and quinoa have gained attention as dietary alternatives due to the growing popularity of gluten-free diets. In this study, we examined the effects of proso millet and quinoa on the human gut microbiota using an in vitro fecal incubation model. Both grains altered alpha diversity metrics, including microbial richness, evenness, and phylogenetic diversity. Beta diversity analysis showed that the proso millet and quinoa treatment groups exhibited distinct clustering patterns compared to the control, highlighting their impact on microbial community structure. Taxonomic analysis showed an increase in beneficial genera, including *Bifidobacterium*, and a decrease in taxa such as *Enterobacteriaceae* and *Flavonifractor*. To assess metabolic changes associated with microbial fermentation, short-chain fatty acid (SCFA) intensities were measured. The intensities of acetic acid, propionic acid, and butyric acid were significantly higher in the proso millet- and quinoa-treated groups compared to the control group. Spearman correlation analysis showed that the abundances of *Bifidobacterium* and *Blautia* were significantly positively associated with SCFA intensities. Furthermore, predicted functional pathway analysis identified enrichment of carbohydrate-related pathways in proso millet and quinoa treatments. Quinoa supplementation led to a broader enhancement of metabolic pathways, including glycolysis/gluconeogenesis, starch and sucrose metabolism, and pentose phosphate pathways, whereas proso millet enriched galactose metabolism, and starch and sucrose metabolism. These findings suggest that proso millet and quinoa influence gut microbial diversity, composition, and function.

## Introduction

Prebiotics are indigestible dietary components that selectively stimulate the growth and activity of beneficial gut microbes, providing significant health benefits to the host (Gibson et al., 2017). Unlike most other dietary components, prebiotics bypass digestion in the upper gastrointestinal tract and reach the colon intact, where they serve as substrates for specific gut microbes. By fostering the growth of health-associated bacteria, prebiotics enhance gut integrity, lower pH levels, and reduce harmful nitrogenous metabolites (Li et al., 2020; Peng et al., 2020). Additionally, through microbial fermentation, they are transformed into bioactive products such as short-chain fatty acids (SCFAs), which support gut health, immune function, and metabolic stability (Esgalhado et al., 2017; Holscher, 2017). This functionality has led to their inclusion as additives or supplements in food products. For example, compounds such as oligosaccharides, resistant starch, inulin, lactulose, pyrodextrins, sugar alcohols, levans, and lactosucrose are incorporated into functional foods to enhance their nutritional and bioactive properties (Cosme et al., 2022; de Paulo Farias et al., 2019). As key components of dietary strategies aimed at preventing dysbiosis and promoting overall health, prebiotics play an essential role in functional nutrition and the development of bioactive food products.

Whole grains, composed of the bran, germ, and endosperm, are nutrient-rich cereals that retain their natural structure during processing, preserving dietary fiber, vitamins, minerals, and bioactive compounds (Joye, 2020). In contrast, refined grains undergo milling, which removes the bran and germ, a process that significantly reduces their levels of non-digestible carbohydrates such as dietary fiber and resistant starch (Slavin et al., 2000). These non-digestible carbohydrates from whole grains pass through the digestive system intact and reach the colon, where they undergo fermentation and provide a source of energy for beneficial gut microbes (Johnstone et al., 2020). With the growing popularity of gluten-free diets, gluten-free whole grains like millet and quinoa are increasingly consumed for their potential health benefits (Woomer and Adedeji, 2021). While whole grain consumption has been linked to reduced risks of chronic diseases, such as cardiovascular disease and diabetes, and improved metabolic stability (Aune et al., 2016), the specific effects of gluten-free whole grains on gut microbiota composition remain understudied.

In this study, we explored the effects of proso millet and quinoa on the human gut microbiota through an in vitro fecal incubation model. By analyzing changes in microbiota composition induced by these gluten-free whole grains, we aimed to evaluate their prebiotic potential and their contribution to gut health. Our study provides a foundation for developing dietary strategies that utilize whole grains to foster microbial balance and promote overall health.

## Materials and Methods

### Study population for fecal microbiome analysis

The study involved 10 healthy Korean participants (5 males and 5 females) with no history of gastrointestinal disorders or antibiotic use within the month prior to the experiment. Fecal samples were collected using sterile cotton swabs and promptly frozen at −80°C for subsequent analysis.

### In vitro anaerobic incubation of fecal samples

Fresh stool samples were transferred into an anaerobic workstation and processed as previously described (Li et al., 2019). Within the anaerobic chamber, samples were homogenized in MiPro medium and filtered through a sterile 985 µm nylon mesh. The resulting homogenate was inoculated into 96-deep well plates to achieve a final fecal concentration of 2.0% (w/v). Commercial whole-grain powders, including proso millet and quinoa obtained from a local market (Korea), were added to the plates at a final concentration of 1.0% (w/v). Fecal suspensions without the whole-grain powder served as controls. The plates were incubated anaerobically at 37°C, and samples were collected at 0 and 24 h.

### 16S rRNA amplicon sequencing and taxonomic analysis

Total genomic DNA was extracted from the incubated stool samples using the DNeasy PowerSoil HTP 96 Kit (Qiagen, Germany). PCR amplification and sequencing were performed according to EMP standard protocols (Caporaso et al., 2012). Using 515F/806R primers, the V4 hypervariable region of the 16S rRNA gene was amplified. The resulting amplicons were pooled and sequenced on the Illumina MiSeq platform (2 × 300 cycles, paired-end). The data were analyzed at the Biopolymer Research Center for Advanced Materials (BRCAM) at Sejong University (Korea). Raw reads were processed using the QIIME 2 software package (version 2020.6) (Bolyen et al., 2019). Sequences were demultiplexed and quality-filtered with the q2-demux plugin. DADA2 was then applied for trimming and denoising (Callahan et al., 2016). The resulting amplicon sequence variants (ASVs) were aligned with MAFFT, and taxonomic classification of each ASV was conducted using the SILVA 132 database (Price et al., 2010; Yilmaz et al., 2014).

### Short-chain fatty acids quantification

To extract metabolites for SCFA analysis, 100 μl of fecal sample was mixed with 25% acetonitrile (1:1, v/v) and vortexed for 20 min. After incubation at 4°C for 1 h, an equal volume of water was added, and the mixtures were centrifuged at 13,000 × g for 10 min. The resulting supernatants were collected for derivatization. Each supernatant (100 µl) was mixed with 25 µl of 50 mM 1-ethyl-3-(3-dimethylaminopropyl) carbodiimide (EDC) in 7% pyridine, followed by 25 µl of 50 mM 3-nitrophenylhydrazine (3-NPH). The mixtures were incubated at 40°C for 30 min, and the reaction was quenched by adding 150 µl of water containing 5% formic acid. Samples were then placed on ice and analyzed using a Vanquish U-HPLC system coupled with an Orbitrap Exploris 120 mass spectrometer (Thermo Fisher Scientific, USA) operated in negative ion mode.

### Prediction of functional metagenomic profiles and KEGG pathway

Functional metagenomic profiles were predicted from 16S rRNA gene sequences using the Phylogenetic Investigation of Communities by Reconstruction of Unobserved States 2 (PICRUSt2, version 2.3.0) bioinformatics software package (Langille et al., 2013). The ASV matrix and representative sequences were used as inputs to estimate Kyoto Encyclopedia of Genes and Genomes (KEGG) ortholog (KO) abundances. To identify significant functional differences between groups, linear discriminant analysis effect size (LEfSe) was applied with an LDA score threshold of > 2.5 and adjusted *p*-value < 0.05 (Segata et al., 2011).

### Data availability

Amplicon sequence data and corresponding metadata are publicly accessible through the EMP data portal on Qiita (https://qiita.ucsd.edu; study ID: 15857), accessed on January 10, 2025.

### Quantification and statistical analysis

Data normality was checked using the Shapiro-Wilk test. For parametric data, comparisons among three or more groups were performed using one-way ANOVA, followed by pairwise comparisons using either the Student’s t-test or Welch's t-test, depending on variance homogeneity. For nonparametric data, comparisons among three or more groups were performed using the Kruskal-Wallis test, followed by pairwise comparisons using the Mann-Whitney U test. Diversity and multivariate analyses were performed in MicrobiomeAnalyst version 2.0 (Lu et al., 2023). The statistical significance of group differences in principal coordinate analysis (PCoA) plots was evaluated using permutational multivariate analysis of variance (PERMANOVA). All *p*-values were adjusted using the Benjamini-Hochberg method to control the false discovery rate.

## Results

### Alpha diversity changes in the gut microbiota induced by proso millet and quinoa

A total of 205,642 sequences (paired-end, Phred score > Q20) were obtained from fecal samples, and these sequences were binned into 470 types of amplicon sequence variant (ASV). One quinoa sample with a sequence count of < 1,000 was excluded from the analysis (Table S1).

**Fig. 1.**
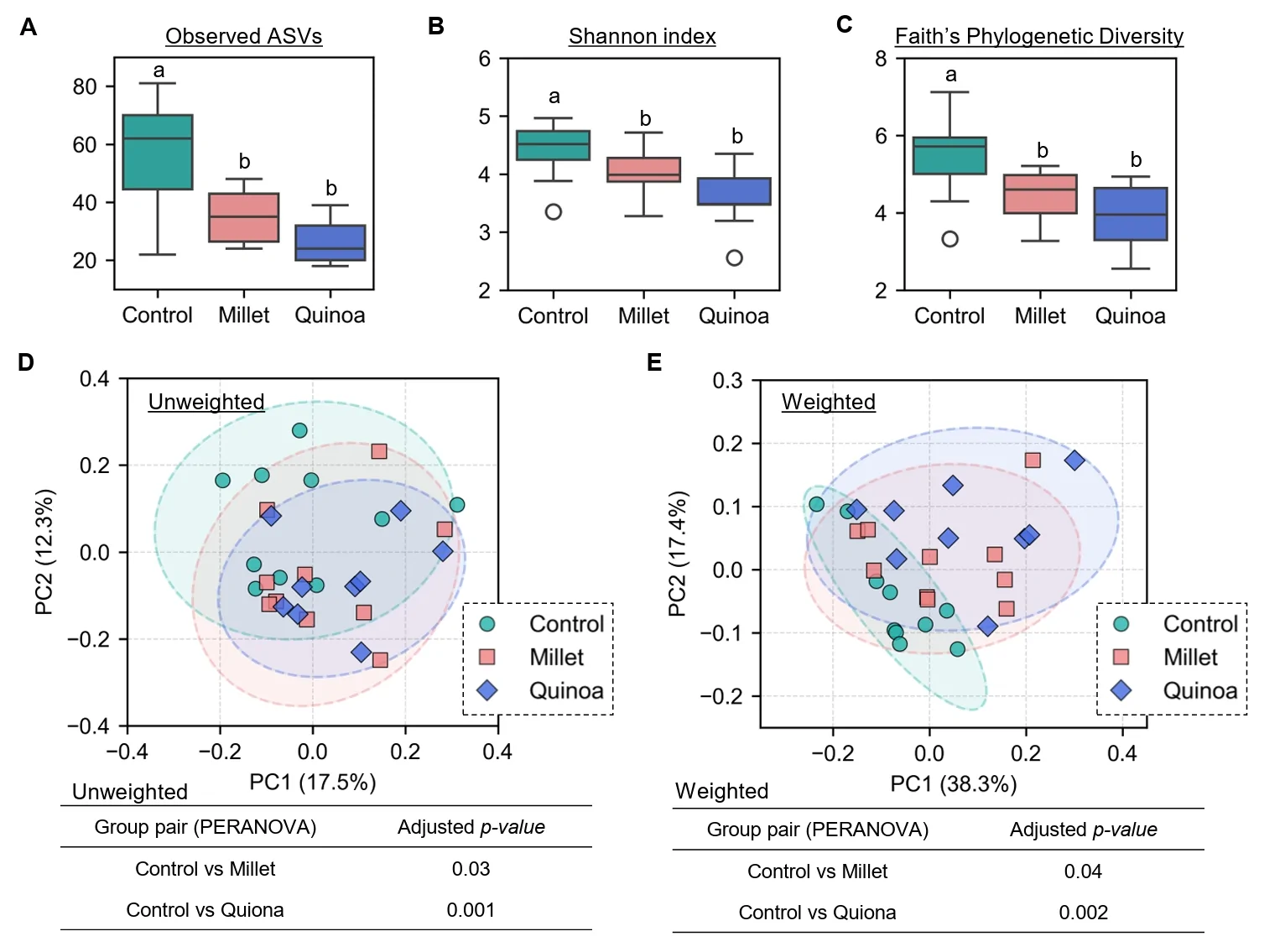
Effects of proso millet and quinoa treatment on gut microbial diversity and structure. Alpha diversity was assessed to evaluate microbial richness, evenness, and phylogenetic diversity using observed amplicon sequence variants (A), Shannon index values (B), and Faith’s phylogenetic diversity (C). Statistical differences between groups are indicated using a compact letter display, where different letters represent significant differences. Microbial structure and composition were analyzed through PCoA based on unweighted (D) and weighted (E) UniFrac distances. Each dot represents an individual sample, and ellipses represent 90% confidence intervals. PERMANOVA results, with adjusted *p*-values, indicate statistical significance among the groups.

We evaluated the effects of the gluten-free whole grains, proso millet (*Panicum miliaceum*) and quinoa (*Chenopodium quinoa*), on the gut microbiota using an in vitro fecal incubation model. Alpha diversity metrics were analyzed to assess microbial richness, evenness, and phylogenetic diversity. Treatment with proso millet and quinoa resulted in a significant reduction in microbial richness and evenness, as indicated by the lower observed amplicon sequence variants (Fig. 1A) and Shannon index values (Fig. 1B) compared to the control group. Faith’s phylogenetic diversity also showed significant decreases (Fig. 1C), suggesting that each grain exerts a distinct influence on the composition and phylogenetic diversity of the gut microbial community.

### Effects of proso millet and quinoa on gut microbiota beta diversity

We performed a beta diversity analysis to evaluate the impact of proso millet and quinoa on gut microbial community composition. Principal coordinates analysis (PCoA) plots, based on unweighted and weighted UniFrac distances, showed distinct clustering for proso millet and quinoa treatments. PCoA analysis using unweighted UniFrac distances, which considers the presence or absence of taxa, revealed significant differences between the control and proso millet groups (PERMANOVA, *p* = 0.03) and between the control and quinoa groups (PERMANOVA, *p* = 0.001; Fig. 1D). Similarly, PCoA analysis using weighted UniFrac distances, which accounts for taxa abundance, indicated significant differences between the control and proso millet groups (PERMANOVA, *p* = 0.04) and between the control and quinoa groups (PERMANOVA, *p* = 0.002; Fig. 1E). However, neither unweighted nor weighted UniFrac analyses showed significant differences between the proso millet and quinoa groups.

### Impact of proso millet and quinoa on key genera in the gut microbiota

**Fig. 2.**
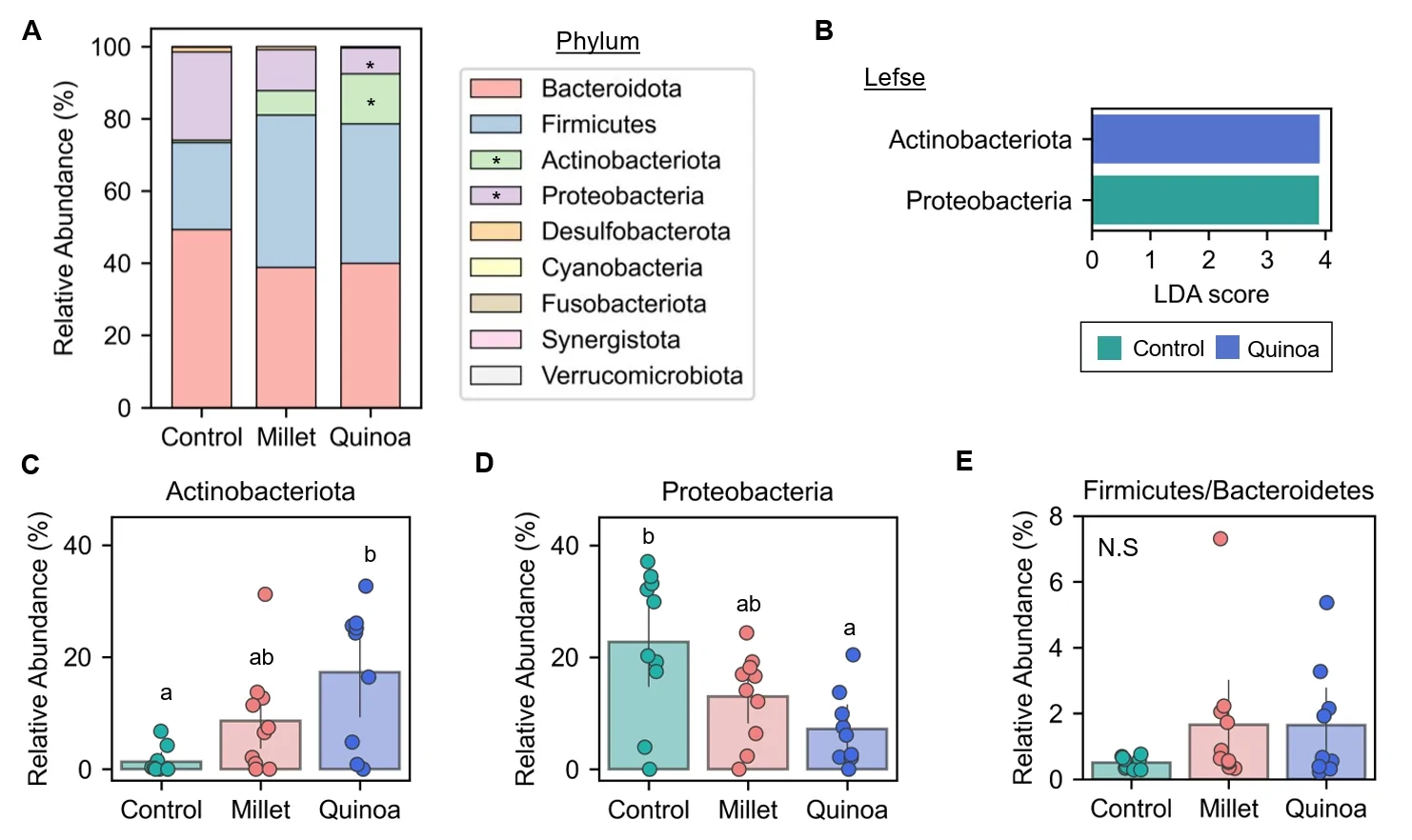
Phylum-level microbial composition changes induced by proso millet and quinoa treatment. (A) The relative abundance of bacterial phyla was assessed across the control, proso millet, and quinoa treatment groups. (B) Differentially abundant taxa among the three groups were identified using LEfSe analysis. The relative abundances of specific phyla, including Actinobacteriota (C) and Proteobacteria (D), were analyzed, along with the Firmicutes-to-Bacteroidota ratio (E). Statistical differences are represented using a compact letter display, where different letters indicate significant differences between groups, and “N.S” denotes no significant difference.

We analyzed the relative bacterial abundance at the phylum level across the control, proso millet, and quinoa groups (Fig. 2A). A total of nine distinct phyla were identified, including Bacteroidota, Firmicutes, Actinobacteriota, Proteobacteria, Desulfobacterota, Cyanobacteria, Fusobacteriota, Synergistota, and Verrucomicrobiota. Differential abundance analysis, using a linear discriminant analysis (LDA) score threshold of > 2.5 and adjusted *p*-values < 0.05, revealed significant differences in Actinobacteriota and Proteobacteria among the groups (Fig. 2B). Actinobacteriota levels were significantly higher in the quinoa group (Fig. 2C), whereas Proteobacteria levels were significantly higher in the control group (Fig. 2D). However, the proso millet group showed no statistically significant differences in Actinobacteriota or Proteobacteria levels compared to either the control or quinoa groups. Additionally, we examined the Firmicutes/Bacteroidota ratio, a key marker of gut homeostasis, and found no statistically significant differences among the groups (Fig. 2E).

**Fig. 3.**
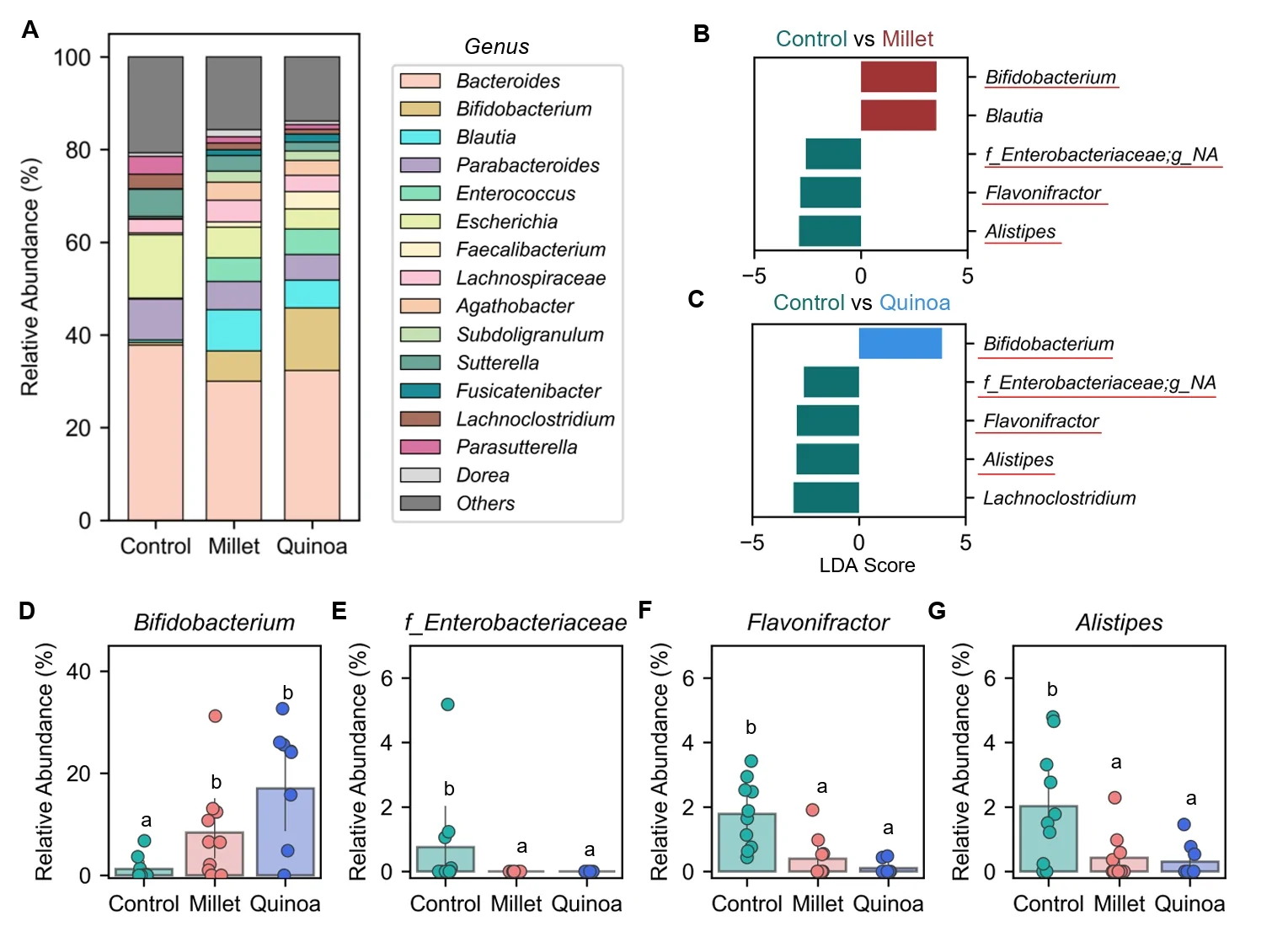
Impact of proso millet and quinoa treatment on gut bacterial genera. (A) The relative abundance of the top 15 bacterial genera was analyzed. Genera with an average abundance greater than 1% across the three groups were included, while the remaining genera were grouped as “Others”. Differentially abundant taxa were identified using LEfSe analysis, comparing control vs. proso millet (B) and control vs. quinoa (C). Labels such as “*f*_” indicate family-level assignments, while “*g_NA*” represents genera not classified at the genus level. The x-axis displays the LDA score. The relative abundances of *Bifidobacterium* (D), f_*Enterobacteriaceae* (E), *Flavonifractor* (F), and *Alistipes* (G) were compared across control and whole grain treatment groups. Statistical differences are represented using a compact letter display, where different letters indicate significant differences between groups.

We further examined the relative abundance of bacterial taxa at the genus level across the control, proso millet, and quinoa groups (Fig. 3A), identifying significant changes using linear discriminant analysis effect size (LEfSe). Compared to the control group, the proso millet group exhibited significant changes in *Bifidobacterium*, *Blautia*, *Enterobacteriaceae*, *Flavonifractor*, and *Alistipes* (Fig. 3B). The quinoa group showed significant differences in *Bifidobacterium*, *Enterobacteriaceae*, *Flavonifractor*, *Alistipes*, and *Lachnoclostridium* (Fig. 3C). We further focused on the four genera that were consistently influenced by both proso millet and quinoa treatments: *Bifidobacterium*, *Enterobacteriaceae*, *Flavonifractor*, and *Alistipes*. When comparing these genera between the proso millet and quinoa groups, no statistically significant differences were observed, suggesting that the two grains exert similar effects on their relative abundance. However, all four genera showed significant differences when comparing the control group to the grain-treated groups. Specifically, the abundance of *Bifidobacterium* increased (Fig. 3D), while *Enterobacteriaceae* (family level, with an unidentified genus), *Flavonifractor* and *Alistipes* exhibited reduced levels in the grain-treated groups relative to the control (Fig. 3E–3G).

### Associations between short-chain fatty acid production and key genera

**Fig. 4.**
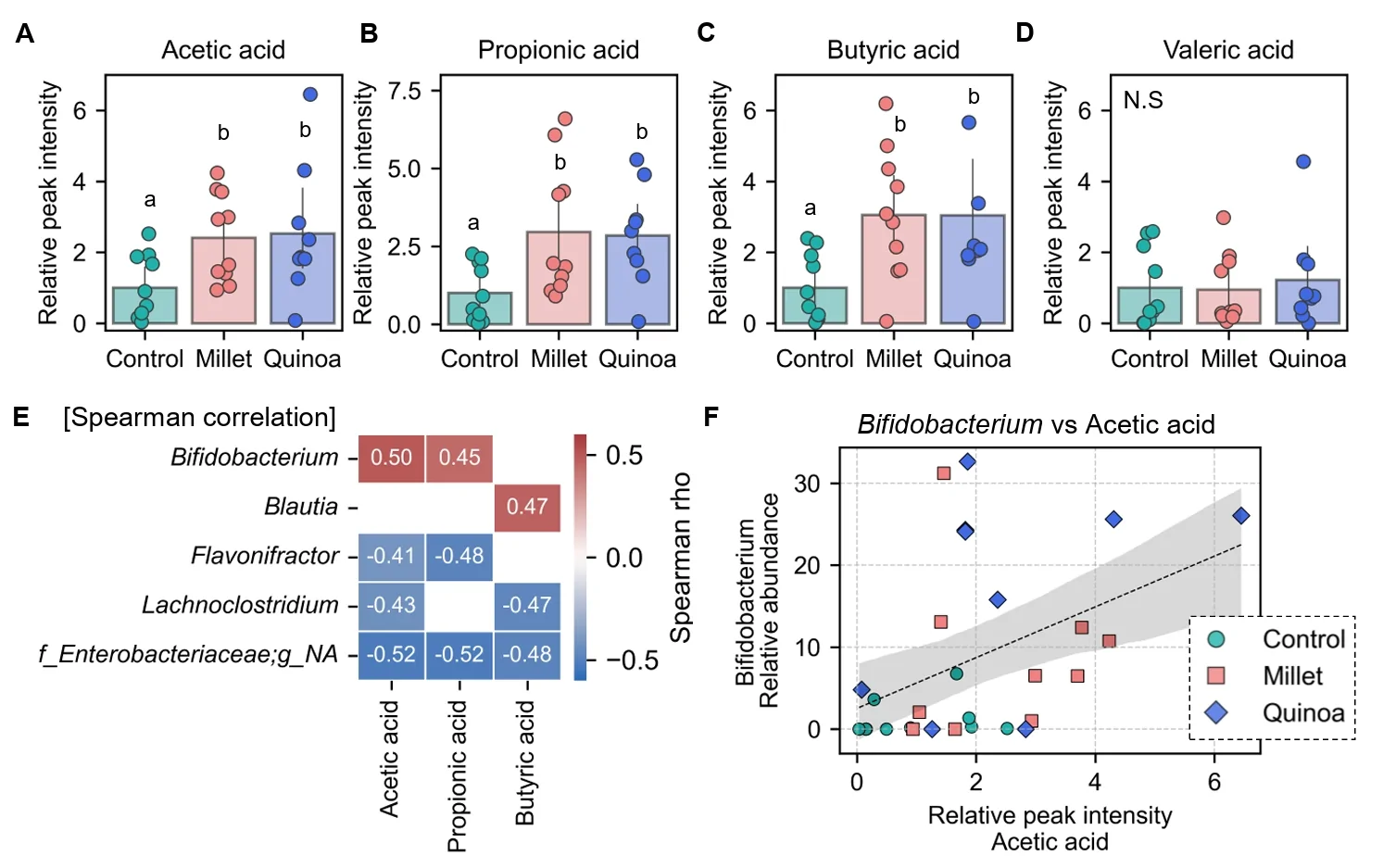
Short-chain fatty acid levels and their associations with key bacterial genera. Short-chain fatty acids (SCFAs) were derivatized and quantified using liquid chromatography–mass spectrometry. The intensities of acetic acid (A), propionic acid (B), butyric acid (C), and valeric acid (D) were measured in fecal samples and compared among the control, proso millet, and quinoa groups. Different letters indicate statistically significant differences among groups (*p* < 0.05). “N.S” denotes no significant difference. (E) Spearman correlation analysis was performed to assess associations between differentially abundant genera (*Bifidobacterium*, *Blautia*, *Flavonifractor*, *Alistipes*, *Lachnoclostridium*, and f_*Enterobacteriaceae*;g_NA) and SCFAs (acetic acid, propionic acid, and butyric acid). Correlation coefficients (ρ) were visualized as a heatmap, with only statistically significant associations (FDR-adjusted *p* < 0.05) displayed. (F) Correlation between *Bifidobacterium* and acetic acid is illustrated in a 2D scatter plot. A linear regression line with a shaded 95% confidence interval is presented.

Proso millet and quinoa treatments altered the gut microbial composition, increasing the abundance of SCFA-producing genera such as *Bifidobacterium* and *Blautia* (Fig. 3A–3C) (Fusco et al., 2023; Holmberg et al., 2024). To determine whether these taxonomic changes translated into metabolic alterations, SCFA concentrations were measured in fecal samples. Four SCFAs, including acetic acid, propionic acid, butyric acid, and valeric acid, were detected (Fig. 4A–4D). Among them, acetic acid, propionic acid, and butyric acid were significantly higher in the proso millet- and quinoa-treated groups compared to the control, whereas valeric acid showed no significant differences among groups.

To investigate the association between SCFA production and microbial composition, Spearman correlation analysis was performed using three SCFAs (acetic acid, propionic acid, and butyric acid) and six genera identified as differentially abundant by LEfSe analysis (*Bifidobacterium*, *Blautia*, *Flavonifractor*, *Alistipes*, *Lachnoclostridium*, and f_*Enterobacteriaceae*;g_NA). Correlation coefficients (rho) with an adjusted *p*-value below 0.05 were visualized in the heatmap (Fig. 4E). The abundance of *Bifidobacterium* showed the strongest positive correlation with acetic acid intensity (rho = 0.50, *p* = 0.028) and was also positively correlated with propionic acid intensity (rho = 0.45, *p* = 0.016). Scatter plot analysis supported this relationship, showing that higher *Bifidobacterium* abundance was associated with increased acetic acid intensity across all groups (Fig. 4F). *Blautia* abundance was positively correlated with butyric acid intensity (rho = 0.47, *p* = 0.010), supporting its involvement in butyrate production (Su et al., 2024). In contrast, *Flavonifractor*, *Lachnoclostridium*, and f_*Enterobacteriaceae*;g_NA abundances were negatively correlated with all three SCFA intensities.

### Predicted changes in gut microbiome functional pathways

**Fig. 5.**
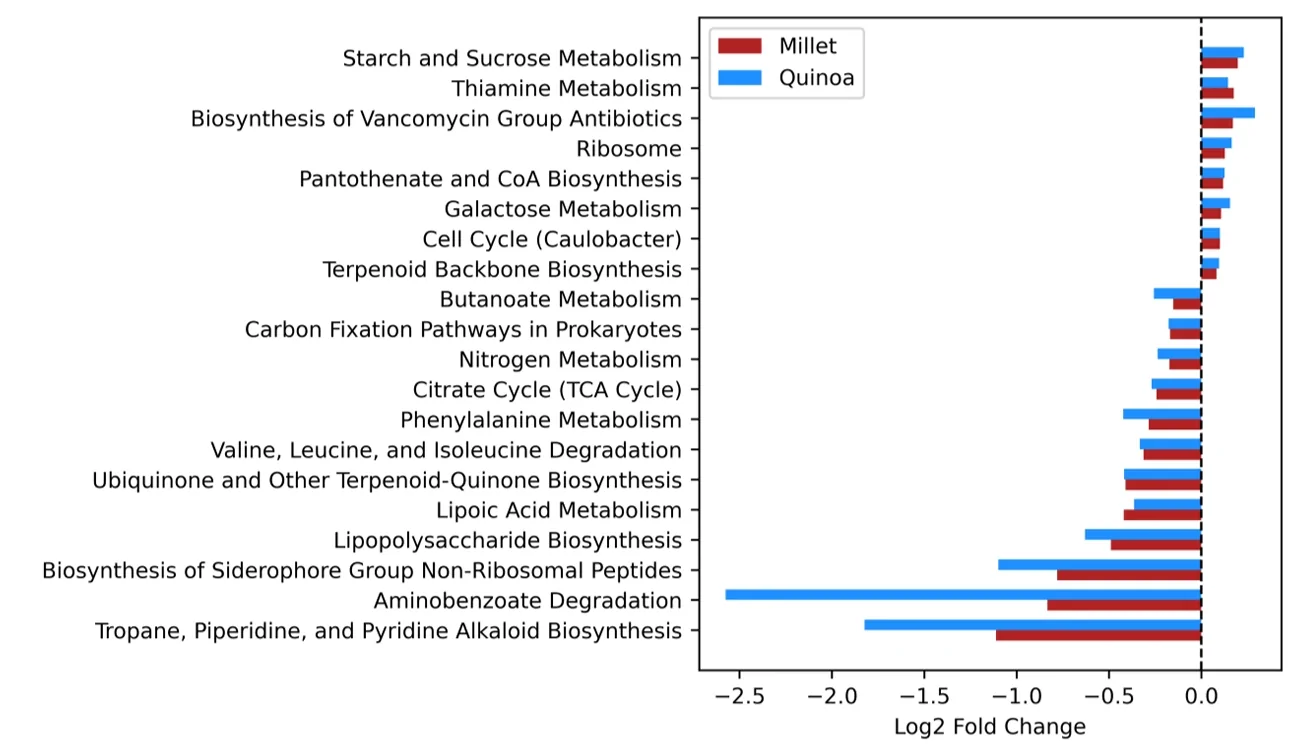
Predicted gut microbiota functional pathway changes induced by proso millet and quinoa treatment. Fold changes (log2) in pathway intensities were calculated relative to the control group. Positive values indicate pathway enrichment, while negative values represent pathway reduction.

We conducted a Phylogenetic Investigation of Communities by Reconstruction of Unobserved States (PICRUSt) analysis to predict functional pathways in the gut microbiome across the whole-grain-treated and control groups. Using 16S rRNA gene sequencing data, PICRUSt inferred the functional potential of microbial communities, focusing on pathways with significant differences identified through LEfSe analysis (*p*-value < 0.05, LDA score > 2.5). The analysis revealed distinct patterns of functional pathway enrichment between the whole-grain-treated groups and the control. The quinoa group showed significant enrichment in 42 pathways compared to the control (Table S2), while the proso millet group enriched 24 pathways (Table S3). Among these, 20 pathways were shared, including those involved in amino acid metabolism, carbohydrate metabolism, lipid metabolism, and the biosynthesis of secondary metabolites (Fig. 5). Since proso millet and quinoa are rich sources of complex carbohydrates, we focused on analyzing carbohydrate-related pathways. The quinoa group demonstrated enrichment in five pathways: glycolysis/gluconeogenesis, pentose phosphate pathway, starch and sucrose metabolism, galactose metabolism, and amino sugar and nucleotide sugar metabolism. The proso millet group enriched two pathways: galactose metabolism and starch and sucrose metabolism. These findings suggest that both proso millet and quinoa enhance microbial functions related to carbohydrate metabolism, but quinoa may have a more diverse impact, potentially improving the gut’s capacity to process dietary fibers and complex carbohydrates efficiently.

## Discussion

In this study involving 10 healthy Korean participants, we investigated the effects of proso millet and quinoa on the human gut microbiota using an in vitro fecal incubation model. Our focus was on their impact on microbial diversity, composition, and functional pathways. Both grains significantly reduced alpha diversity metrics, including microbial richness, evenness, and phylogenetic diversity, compared to the control group, indicating distinct effects on microbial community structure (Fig. 1A–1C). Beta diversity analysis revealed a clear separation between the grain-treated and control groups, with no significant differences observed between proso millet and quinoa (Fig. 1D and 1E). At the taxonomic level, quinoa increased the abundance of Actinobacteriota, while Proteobacteria was more abundant in the control group (Fig. 2). Both grains consistently influenced key genera, increasing *Bifidobacterium* while reducing *Enterobacteriaceae* (family level, with an unidentified genus), *Flavonifractor* and *Alistipes*, suggesting common prebiotic-like effects (Fig. 3). Additionally, levels of beneficial metabolites, including acetic acid, propionic acid, and butyric acid, were elevated in fecal samples following whole grain treatment (Fig. 4). Predicted functional pathway analysis using PICRUSt showed that carbohydrate-related pathways were enriched in both proso millet and quinoa groups compared to the control (Fig. 5). These findings highlight the potential of proso millet and quinoa to modulate the gut microbiome and enhance carbohydrate metabolism-related activities.

To function as a prebiotic, a dietary component needs to resist digestion and absorption in the gastrointestinal tract, allowing it to reach the gut microbiota intact. Whole grains, such as quinoa and millet, are abundant in dietary fiber, a non-digestible carbohydrate that bypasses digestion in the small intestine and supports microbial fermentation in the colon. Quinoa contains 7–27% dietary fiber (Khan et al., 2024; Nowak et al., 2016; Pulvento et al., 2012), while millet provides 13–14% (Jayawardana et al., 2019; Khan et al., 2024), reflecting their substantial fiber content. We quantified the fraction of starch resistant to hydrolysis by human digestive enzymes in the proso millet and quinoa used in this study. An in vitro digestion model simulating oral, gastric, and small intestinal conditions was employed to measure the amount of undigested starch remaining after enzymatic treatment (Brodkorb et al., 2019). The analysis showed that, after simulated digestion, 22.54 g of starch per 100 g of raw proso millet and 5.21 g per 100 g of raw quinoa remained undigested (Fig. S1). These residual starches may act as fermentable substrates for colonic microbiota, contributing to prebiotic effects. Although our findings indicate that proso millet and quinoa exhibit prebiotic potential, the in vitro fecal incubation model used in this study did not simulate digestive processes. Incorporating simulated digestion steps prior to incubation would improve the physiological relevance and should be considered in further studies.

This study found that quinoa and proso millet influence gut microbiota composition, with a notable increase in the relative abundance of *Bifidobacterium* following treatment with these whole grains (Fig. 3D). Since *Bifidobacterium* utilizes various dietary fibers, including fructans, arabinoxylan, β-glucan, and resistant starch (Wang et al., 2022), the dietary fibers in the whole grains used in this study may support its growth and metabolic activity. Importantly, *Bifidobacterium* contributes to the production of SCFAs, which are associated with anti-inflammatory effects (Horiuchi et al., 2020; Yoshii et al., 2019). These findings align with established evidence that dietary fiber intake can modulate immune responses and reduce the risk of chronic diseases (Krishnamurthy et al., 2012; Li and Ma, 2024; Partula et al., 2020). While further research is needed to elucidate the specific mechanisms, this study provides a foundation for understanding the potential health benefits of whole grains.

Proso millet and quinoa treatments significantly increased the abundances of SCFA-producing genera, particularly *Bifidobacterium* and *Blautia*. Corresponding to these taxonomic changes, levels of acetic acid, propionic acid, and butyric acid were elevated in incubated fecal samples. *Bifidobacterium* is recognized for fermenting dietary carbohydrates into acetate through the bifid shunt pathway (Schöpping et al., 2021). In this study, *Bifidobacterium* abundance was the strongest positive correlation with acetic acid intensity (rho = 0.50, *p*-value = 0.028) among the detected SCFAs, supporting its role as an acetate producer in the gut microbiota (Fig. 4E). *Bifidobacterium* abundance was also associated with butyric acid intensity (rho = 0.38, *p*-value = 0.070). Although *Bifidobacterium* does not directly synthesize butyrate, the acetate it produces can be utilized by butyrate-producing bacteria, thereby indirectly contributing to butyric acid production through metabolic cross-feeding (Xiao et al., 2024). While correlation analysis cannot establish causality, these associations suggest that proso millet and quinoa treatments may promote beneficial metabolic interactions within the gut microbiota.

PICRUSt predicts putative metabolite functions based on 16S rRNA sequencing data (Langille et al., 2013). While not directly related to carbohydrate metabolism, this study observed a significant reduction in the aminobenzoate degradation pathway following whole grain treatment, with a 5.96-fold decrease in the quinoa group and a 1.78-fold decrease in the proso millet group compared to the control (Fig. 5). This reduction is likely linked to changes in microbial taxa that utilize aminobenzoate (PABA) as a substrate (Engevik et al., 2019). Proteobacteria, which are known to degrade PABA (Zhao et al., 2022; Ziegler et al., 1987), declined in response to whole grain treatment (Fig. 2D), while the abundance of folate-synthesizing bacteria, such as *Bifidobacterium*, which use PABA as a precursor for folate biosynthesis (D’Aimmo et al., 2023), increased (Fig. 3D). These microbial compositional changes suggest that whole grain treatment may shift PABA utilization from degradation to biosynthesis. However, since PICRUSt predicts metabolic pathways based on 16S rRNA data, the accuracy of these pathway predictions is inherenthly limited. PICRUSt inferences rely on gene annotations, and previous research has shown that microbial gene annotations may be inaccurate (Langille, 2018; Radivojac et al., 2013). In addition, annotated genes may not be transcribed or translated, reducing the functional relevance of their predicted roles. To further clarify these changes, future studies should employ shotgun metagenomic sequencing, which enables a comprehensive analysis of microbial genetic content and functional pathways (Li et al., 2023; Quince et al., 2017). While this study focused primarily on microbial composition, these findings suggest that whole grain treatment may influence gut microbial metabolic processes.

When whole grains were introduced during fecal incubation, changes were observed in four key genera: *Bifidobacterium* increased, while *Enterobacteriaceae*, *Flavonifractor*, and *Alistipes* decreased (Fig. 3D–3G). *Bifidobacterium*, a beneficial genus known for producing SCFAs such as acetate and lactate, supports gut health by lowering pH (Usta-Gorgun and Yilmaz-Ersan, 2020) and inhibiting the growth of pathogenic bacteria (Hütt et al., 2006), highlighting the prebiotic potential of these grains. The reduction in *Enterobacteriaceae*, a family that includes opportunistic pathogens linked to inflammation and dysbiosis (Janda and Abbott, 2021; Lemons et al., 2024), suggests that proso millet and quinoa may help restore microbial balance. The decrease in *Flavonifractor*, which is associated with pro-inflammatory metabolites (Coello et al., 2021; Eicher and Mohajeri, 2022), indicates a reduction in inflammation-related microbes. Similarly, the decline in *Alistipes*, which has been linked to various disease states despite its context-dependent roles (Fu et al., 2024; Parker et al., 2020), suggests potential benefits. While predicting gut health solely based on changes in microbial abundance carries the risk of overinterpretation, these findings suggest that whole grains have the potential to shift the gut environment toward improved microbial balance and overall gut health.

Several studies have examined the effects of quinoa, millet, and their derived molecules on gut microbiota composition and short-chain fatty acid production under in vitro fecal incubation conditions (Gullón et al., 2016; Zeyneb et al., 2021). Consistent with these findings, our study demonstrated a selective enrichment of beneficial taxa, particularly *Bifidobacterium*, along with increased concentrations of acetic and butyric acids. These observations reinforce the prebiotic potential of gluten-free whole grains such as proso millet and quinoa, highlighting their capacity to promote beneficial microbial shifts and enhance metabolic activity within the gut ecosystem. In microbiome research, rigorous validation is essential because microbial responses to dietary interventions are shaped by host genetics, geography, habitual diet, and baseline microbiota composition (Borrello et al., 2022; Leshem et al., 2020). Without rigorous validation, observed effects may remain population-specific and lack generalizability, thereby limiting their translational relevance. Our study validates the effects of proso millet and quinoa on gut microbiota composition and metabolic activity. By confirming consistent microbial and metabolic responses in a Korean population, these findings reinforce the robustness of their prebiotic effects and support their broader applicability across diverse populations.

The small sample size (n = 10) is a major limitation of this study and indicates the need for future investigations with larger cohorts to enhance the reliability of the results. In addition to the limited sample size, the fecal samples analyzed were geographically confined to Korean participants, highlighting the necessity of validating these findings in populations with broader geographical and ethnic diversity. However, previously reported in vitro studies conducted in other regions, including China and Europe (Gullón et al., 2016; Zeyneb et al., 2021), have observed increases in *Bifidobacterium* abundance and enhanced SCFA production following whole grain interventions, which are consistent with our findings. Furthermore, an in vivo study reported an increase in *Bifidobacterium* abundance in mice following millet porridge supplementation (Chen et al., 2022), aligning with our observations. These consistent findings across different experimental models support the prebiotic potential of proso millet and quinoa.

Our findings highlight the potential of proso millet and quinoa as dietary components that modulate the gut microbiome and enhance microbial functions associated with gut health. The observed changes in microbial diversity, composition, and functional pathways suggest that these whole grains influence key aspects of gut microbial ecology, including an increase in beneficial genera such as *Bifidobacterium* and a reduction in potentially harmful taxa such as *Enterobacteriaceae*. Taken together, proso millet and quinoa act as gut-modulating agents and may serve as effective components in broader nutritional interventions aimed at promoting gut health and maintaining microbial balance.

## Supplementary Information

Table S1. Sequencing information

Table S2. Putative functional pathways enriched in the control and quinoa

Table S3. Putative functional pathways enriched in the control and proso millet

Fig. S1. Total and resistant starch contents of proso millet and quinoa. Starch contents are expressed as grams per 100 g dry weight. Total starch was measured using the Total Starch Assay Kit (K-TSTA, Megazyme). Resistant starch was quantified from the undigested fraction remaining after simulated oral, gastric, and intestinal digestion, followed by dialysis. Starch in the undigested fraction was hydrolyzed and measured colorimetrically using glucose oxidase-peroxidase reagent.
