# Supplementary material for: Prebiotic potential of proso millet and quinoa: Effects on gut microbiota composition and functional metabolic pathways
Source: J Microbiol. 2025 Jul 31;63(7):e2503002. doi: 10.71150/jm.2503002 (PMC13577089; doi:10.71150/jm.2503002)
Supplement: Supplementary file 1 [file jm-2503002-Supplementary-Fig-S1.pdf]

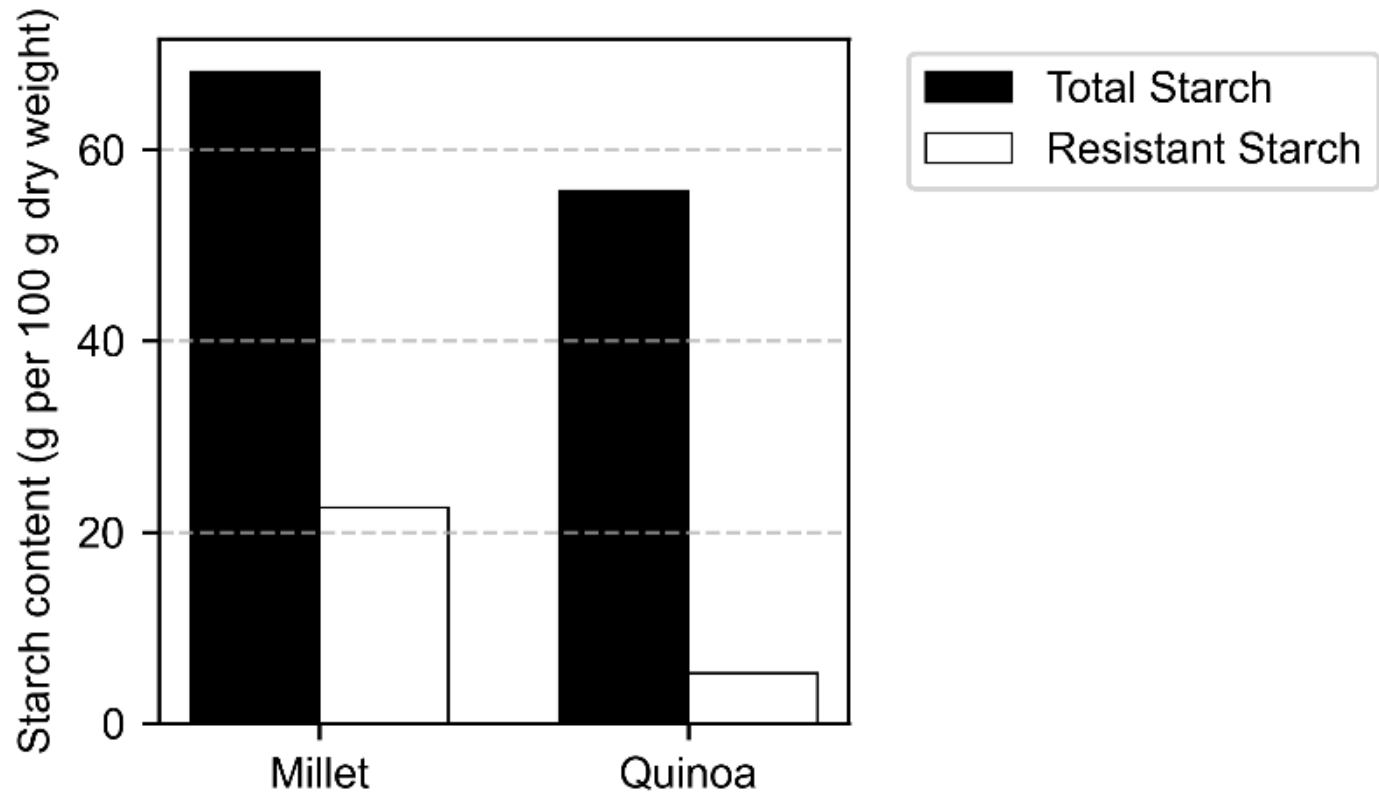

Fig. S1. Total and resistant starch contents of proso millet and quinoa. Starch contents are expressed as grams per 100 g dry weight. Total starch was measured using the Total Starch Assay Kit (K-TSTA, Megazyme). Resistant starch was quantified from the undigested fraction remaining after simulated oral, gastric, and intestinal digestion, followed by dialysis. Starch in the undigested fraction was hydrolyzed and measured colorimetrically using glucose oxidase-peroxidase reagent.
