# Supplementary material for: Prebiotic potential of proso millet and quinoa: Effects on gut microbiota composition and functional metabolic pathways
Source: J Microbiol. 2025 Jul 31;63(7):e2503002. doi: 10.71150/jm.2503002 (PMC13577089; doi:10.71150/jm.2503002)
Supplement: Supplementary file 2 [file jm-2503002-Supplementary-Table-S1.pdf]

Table S1. Sequencing information

Group	Control	Proso millet	Quinoa
Number of samples	10	10	9
Total number of sequences (seqs)	142,344	36,372	26,926
Mean number of sequences ± SD[a]	14,234 ± 10,283	3,637 ± 1,513	2,992 ± 1,368
Number of observed ASV[b]	313	152	112

[a]SD, Standard Deviation; [b]ASV, Amplicon Sequence Variant.
