# Supplementary material for: Prebiotic potential of proso millet and quinoa: Effects on gut microbiota composition and functional metabolic pathways
Source: J Microbiol. 2025 Jul 31;63(7):e2503002. doi: 10.71150/jm.2503002 (PMC13577089; doi:10.71150/jm.2503002)
Supplement: Supplementary file 3 [file jm-2503002-Supplementary-Table-S2.pdf]

Table S2. Putative functional pathways enriched in the control and quinoa

Putative Functional Pathway	Enriched Group	LEfSe Score	p-value
Tyrosine metabolism	Control	2.54	0.0082
Biosynthesis of siderophore group non-ribosomal peptides	Control	2.6	0.0191
Valine, leucine, and isoleucine degradation	Control	2.65	0.0004
Two-component system	Control	2.7	0.0126
Ubiquinone and other terpenoid-quinone biosynthesis	Control	2.75	0.0413
Phenylalanine metabolism	Control	2.75	0.0041
Butanoate metabolism	Control	2.78	0.0019
Nitrogen metabolism	Control	2.78	0.0041
Glyoxylate and dicarboxylate metabolism	Control	2.8	0.0082
Carbon fixation pathways in prokaryotes	Control	2.82	0.0233
Nitro toluene degradation	Control	2.82	0.0233
Aminobenzoate degradation	Control	2.85	0.0009
Flagellar assembly	Control	2.9	0.0052
Bacterial chemotaxis	Control	2.95	0.0233
Citrate cycle (TCA cycle)	Control	2.98	0.0019
Lipoic acid metabolism	Control	3.1	0.0284
Synthesis and degradation of ketone bodies	Control	3.16	0.001
Tropane, piperidine, and pyridine alkaloid biosynthesis	Control	3.2	0.0026
Lipopolysaccharide biosynthesis	Control	3.27	0.0065
Pyrimidine metabolism	Quinoa	2.51	0.0191
Nucleotide excision repair	Quinoa	2.61	0.0032
DNA replication	Quinoa	2.64	0.0082
Terpenoid backbone biosynthesis	Quinoa	2.64	0.0191
Nicotinate and nicotinamide metabolism	Quinoa	2.66	0.0025
One-carbon pool by folate	Quinoa	2.68	0.0102
Homologous recombination	Quinoa	2.71	0.0156
Mismatch repair	Quinoa	2.72	0.0191
Cell cycle (Caulobacter)	Quinoa	2.73	0.0233
Glycolysis / Gluconeogenesis	Quinoa	2.75	0.0156
Amino sugar and nucleotide sugar metabolism	Quinoa	2.76	0.0494
Bisphenol degradation	Quinoa	2.84	0.0306
Thiamine metabolism	Quinoa	2.85	0.0494
Pantothenate and CoA biosynthesis	Quinoa	2.86	0.0012
Pentose phosphate pathway	Quinoa	2.89	0.0494
Galactose metabolism	Quinoa	2.9	0.0413
Aminoacyl-tRNA biosynthesis	Quinoa	2.91	0.0126
Peptidoglycan biosynthesis	Quinoa	2.92	0.0191
Ribosome	Quinoa	2.92	0.0082
D-Glutamine and D-glutamate metabolism	Quinoa	2.99	0.0126
Starch and sucrose metabolism	Quinoa	3.03	0.0025
Secondary bile acid biosynthesis	Quinoa	3.39	0.0156
Biosynthesis of vancomycin group antibiotics	Quinoa	3.45	0.0005
