# Supplementary material for: Prebiotic potential of proso millet and quinoa: Effects on gut microbiota composition and functional metabolic pathways
Source: J Microbiol. 2025 Jul 31;63(7):e2503002. doi: 10.71150/jm.2503002 (PMC13577089; doi:10.71150/jm.2503002)
Supplement: Supplementary file 4 [file jm-2503002-Supplementary-Table-S3.pdf]

Table S3. Putative functional pathways enriched in the control and proso millet

Putative Functional Pathway	Enriched Group	LEfSe Score	*p*-value
Biosynthesis of siderophore group non-ribosomal peptides	Control	2.55	0.0494
Aminobenzoate degradation	Control	2.56	0.0306
Valine, leucine, and isoleucine degradation	Control	2.61	0.0065
Butanoate metabolism	Control	2.64	0.0233
Phenylalanine metabolism	Control	2.64	0.0191
Nitrogen metabolism	Control	2.71	0.0494
Propanoate metabolism	Control	2.74	0.0451
Ubiquinone and other terpenoid-quinone biosynthesis	Control	2.8	0.0284
Carbon fixation pathways in prokaryotes	Control	2.88	0.0082
Citrate cycle (TCA cycle)	Control	2.99	0.0025
Tropane, piperidine, and pyridine alkaloid biosynthesis	Control	3.03	0.0052
Lipoic acid metabolism	Control	3.11	0.0284
Lipopolysaccharide biosynthesis	Control	3.24	0.0191
Glycosaminoglycan degradation	Control	3.28	0.0284
Terpenoid backbone biosynthesis	Proso millet	2.55	0.0494
Cell cycle (Caulobacter)	Proso millet	2.66	0.0284
Ribosome	Proso millet	2.74	0.0413
RNA polymerase	Proso millet	2.79	0.0233
Pantothenate and CoA biosynthesis	Proso millet	2.81	0.0156
Galactose metabolism	Proso millet	2.82	0.0156
Beta-lactam resistance	Proso millet	2.82	0.0413
Thiamine metabolism	Proso millet	2.95	0.0041
Starch and sucrose metabolism	Proso millet	2.96	0.0102
Biosynthesis of vancomycin group antibiotics	Proso millet	3.12	0.0065
